# Pretreatment with Ginseng Fruit Saponins Affects Serotonin Expression in an Experimental Comorbidity Model of Myocardial Infarction and Depression

**DOI:** 10.14336/AD.2016.0729

**Published:** 2016-12-01

**Authors:** Mei-Yan Liu, Yan-Ping Ren, Li-Jun Zhang, Jamie Y. Ding

**Affiliations:** ^1^Department of Cardiology, Beijing Anzhen Hospital, Capital Medical University, Beijing 100029, China; ^2^Department of Geriatric-Cardiovascular Diseases, the First Hospital of Xi’an Jiaotong University, Xi'an, Shaanxi 710061, China; ^3^Dongzhimen Hospital, Beijing University of Chinese Medicine, Beijing 100029, China; ^4^Wayne State University School of Medicine, Detroit, Michigan 48201, USA

**Keywords:** serotonin, ginseng, myocardial infarction, depression, brain

## Abstract

We previously demonstrated that serotonin (5-HT) and 5-HT_2A_ receptor (5-HT_2A_R) levels in platelets were up- or down-regulated after myocardial infarction (MI) associated with depression. In this study, we further evaluated the effects of pretreatment with ginseng fruit saponins (GFS) on the expression of 5-HT and 5-HT_2A_R in MI with or without depression. Eighty Sprague-Dawley (SD) rats were treated with saline and GFS (n=40 per group). The animals were then randomly divided into four subgroups: sham, MI, depression, and MI + depression (n=10 per subgroup). Protein levels of 5-HT and 5-HT_2A_R in the serum, platelets and brain tissues were determined with ELISA. The results demonstrated that serum 5-HT levels was significantly increased by GFS pretreatment in all subgroups (except the sham subgroup) when compared with saline-treated counterparts (p<0.01). In platelets, GFS pretreatment significantly increased 5-HT levels in all subgroups when compared with their respective saline-treated counterparts (p<0.01). Brain 5-HT levels also declined with GFS pretreatment in the MI-only and depression-only subgroups (p<0.05 *vs.* saline pretreatment). With respect to 5-HT_2A_R levels, platelet 5-HT_2A_R was decreased in GFS pretreated MI, depression and MI + depression subgroups (p<0.01 *vs.* saline pretreatment). Similarly, brain 5-HT_2A_R levels decreased in all four subgroups pretreated with GFS (p<0.01 *vs.* saline pretreatment). We conclude that GFS plays a clear role in modulating 5-HT and 5-HT_2A_R expressions after MI and depression. Although the effects of GFS on brain 5-HT remain to be elucidated, its therapeutic potential for comorbidities of acute cardiovascular events and depression appears to hold much promise.

Coronary heart disease and clinical depression are commonly associated; patients worldwide have more often than not experienced symptoms of depression while recovering from myocardial infarction (MI). The incidence of coronary heart disorder among depression cases ranges from 5% to 50% depending on the location and scope of study [1-4]. There is a substantial amount of evidence that depression is an independent risk factor for coronary heart disease, while patients with heart disease also have a high risk of depression [5]. Patients with multiple cardiovascular disease-associated depression and/or anxiety disorder have been recognized at home and abroad [6,7]. In 2013, a survey of general hospitals in five major Chinese cities found 2123 such cases. The current prevalence of depression in China is 10.55% with a lifetime prevalence is 13.75% while anxiety disorders have a current prevalence of 7.77% and a lifetime prevalence of 8.53% [8]. Studies show that depression and/or anxiety can increase the risk of cardiovascular disease by a factor of 1.5 to 2.7 [9]. The specific mechanism is unclear, but depression and cardiovascular disease may reinforce each other in comorbidity [10,11]. Thrombosis and increased platelet activity are important pathological features of atherosclerosis and acute coronary events, but they are also found in patients with depression. Platelet activation may play an important role in either MI or depression, and increase the vulnerability of depressed MI patients to cardiac events due to endothelium damage, dyslipidemia and elevation in circulating substances such as thromboxane [12,13].

Of the factors that could affect both MI and depression, the neurotransmitter serotonin (5-hydroxytryptamine, 5-HT) is one of the more intriguing culprits. Platelets contain approximately 99% of all the 5-HT in the bloodstream and have been known to release 5-HT at vascular injury sites [14]. 5-HT is critical to thrombus formation, platelet aggregation, vascular wall repair and proliferation of endothelial cells, but elevated 5-HT in blood is associated with increased risk of cardiac events in MI patients [15]. 5-HT imbalance is also associated with depression. In depression, platelet reactive activity is elevated with platelets presenting increased levels of the serotonin 5-HT_2A_ receptor (5-HT_2A_R) [16]. Therefore, 5-HT may be involved in the pathogenesis of comorbid coronary heart disease and depression by affecting both platelet function and mental state [17].

Clinical work suggests that various traditional Chinese medicine (TCM) remedies may affect this interaction [18]. Ginseng fruit saponins (GFS) extracted from the ginseng fruit are particularly promising candidates for effective therapeutic interventions [19]. Ginseng, the rhizome of the *Panax ginseng* plant, has been used for thousands of years in TCM for a multitude of purposes. Indeed, the word “panax” is derived from “panacea,” meaning “cure-all” in Greek [20]. Ginsenosides, the active ingredients of GFS, are triterpene saponins composed of a dammarane skeleton with various sugars attached at the C-3 and C-20 positions [21]. Over 30 different ginsenosides in GFS have been identified and classified into two categories: 20(S)-protopanaxadiols (PPD) and 20(S)-protopanaxatriols (PPT). PPTs differ from PPDs in that they possess an additional hydroxyl moiety or sugar residue at the C6 position [22]. It has been reported that GFS have beneficial effects on both the nervous and circulatory systems [23,24].

In the present study, we aimed to establish the effects of GFS pretreatment on the comorbidity of MI and depression by quantifying levels of 5-HT and 5-HT_2A_R in the serum, platelets and brain. Platelets were chosen because their demonstrated similarity to neurons suggested a potential for diagnostic use, considering that a blood test is much less invasive and expensive than nervous tissue biopsy [16]. 5-HT_2A_R was not measured in the serum because it is membrane-bound and not found in serum.

## MATERIALS AND METHODS

### Subjects

In this study, we used 80 Sprague Dawley (SD) rats, both male and female, weighing 180-200 grams (Pharmaceutical Base, Jiangsu Province). The rats were randomly divided into two pre-treated groups with GFS (Jilin Ji’an Yisheng Pharmaceutical Co. Ltd.) or placebo saline (n=40 per group). After pretreatment (4 weeks), both groups were further divided into four subgroups (n=10 per subgroup): 1) control/sham operation without MI and depression; 2) depression; 3) MI; 4) combined MI and depression (MI + depression). Animals were then sacrificed after 3 days to observe the effects of GFS on levels of 5-HT and 5-HT_2A_R in the rat serum, platelets, and brain tissues.

### Pretreatment

The GFS-pretreated rats received GFS by gavages at 20 mg/kg dissolved in 2.5 ml saline once a day for 4 weeks while the saline-pretreated rats received an equivalent volume of placebo saline for the same period.

### Various Pathological States

After the 4 weeks of pretreatment, 4 different pathological conditions were induced. 1) MI was performed with Akbay’s approach [28]. Rats, anesthetized with ketamine (40 mg/kg) and xylazine (1 mg/kg) via intra-muscular injection, were placed in the supine position. After disinfecting, the thorax was opened in the fourth intercostal space. The left anterior descending artery (LAD) was cauterized at the midpoint through the starting point and the cardiac apex. After cauterization, the air in the thorax was squeezed out by the fore finger and the thorax was closed with the suture. 2) Depression was induced with the Modified Forced Swimming Test (FST) described previously by Porsolt [25-27]. Rats were plunged individually in a cylinder (40 cm height, 20 cm diameter) containing 30 cm water maintained at 25°C. After 15 min in the cylinder, they were removed and allowed to dry for 15 min in a heated enclosure (32°C) before returning to their individual cages. This procedure involved long periods of immobility in the water (10-12 min total) and hypoactivity for 30 min. After 24 h, the FST was repeated except this time, the rats were placed in the cylinder for only 5 min. 3) MI + Depression was induced by first performing MI surgery then FST 3 days after the surgery, following the procedures described above. 4) Sham (no induction of MI and depression) rats were anesthetized but did not receive thoracotomy or cauterization of the left anterior artery. FST was also not performed.

### ELISA

Protein levels were determined using ELISA kits specific for 5-HT (product #: MEXN-M136) and 5-HT_2A_R (product #: MEXN-M142) obtained from Shanghai Meixuan Biological Science and Technology Ltd. Platelet-rich plasma (PRP) was prepared by centrifuging blood for 15 minutes at room temperature, 2,000 RPM, within 30 minutes of blood collection [29]. Platelets were then separated by centrifuging PRP for 10 minutes at 4 °C, 2,100 RPM [30]. 5-HT levels were measured using the standardized ELISA method (Instructions for use: Serotonin ELISA. IB89546 [DB/OL]; http://www.ibl-america.com/pdf/elisa/IB89546.pdf) [31, 32]. 5-HT_2A_R levels were measured according to Jurado’s receptor assay [33]. The unit used in all cases was pg/ml.

### Statistical Analysis

We used SPSS 19.0 for all data processing and analysis. Data was represented as mean ± standard deviation (SD). Comparisons between two subgroups were assessed using the independent t-test. P values less than 0.05 were considered statistically significant.

**Figure 1.**
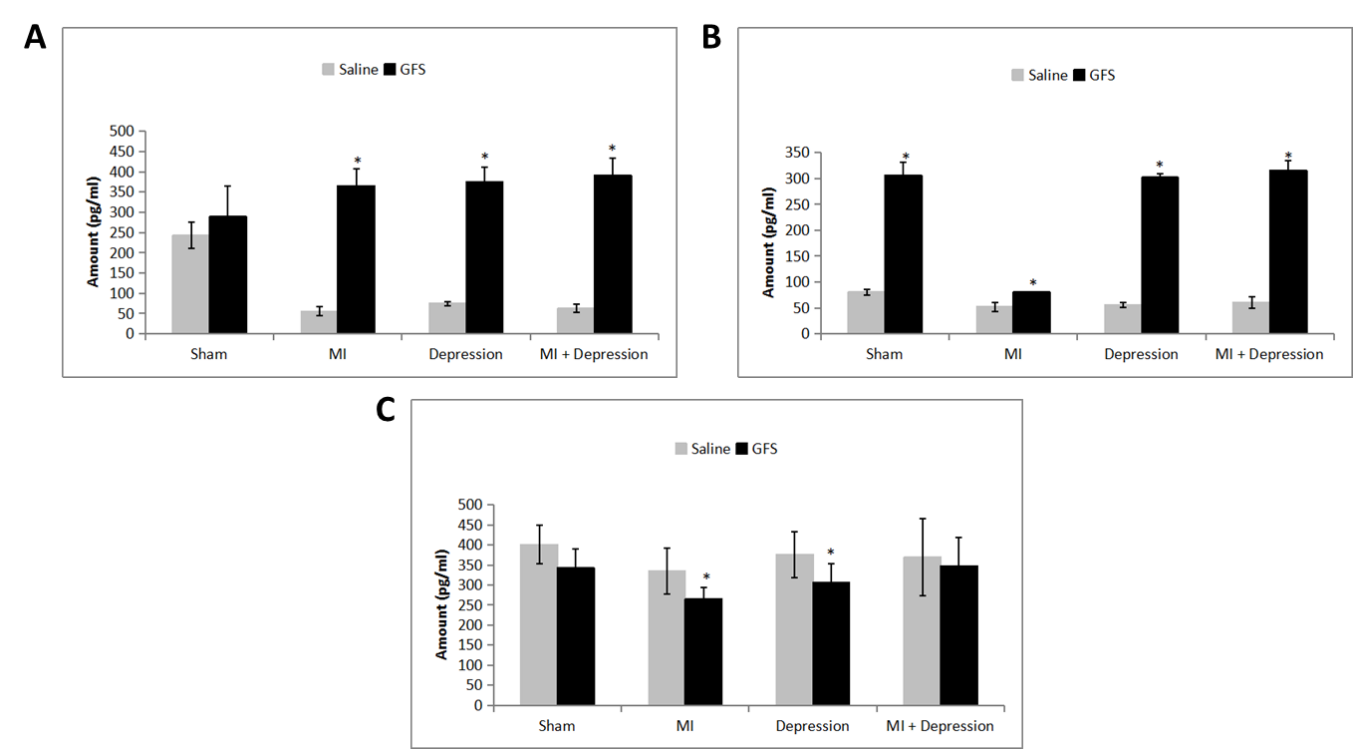
**Animals were first randomly divided into two groups: saline and GFS (n=40 per group).** The rats were pretreated with GFS (20 mg/kg) or with an equivalent volume of saline once daily via oral gavage for a period of 4 weeks. Rats were then equally divided randomly into four subgroups (n=10 per subgroup) and the appropriate surgeries and tests were performed: Sham, MI, Depression, MI + Depression. After 3 days, animals were sacrificed and 5-HT levels measured in the serum, platelets, and brain tissues using an ELISA kit. Data are presented as mean ± SD. **A**) Quantification of 5-HT level in serum. As compared with saline-treated animals, GFS pretreatment increased 5-HT levels in the sham group although it did not achieve significance (*p*=0.184). However, there was a significant increase in 5-HT levels in the GFS-pretreated MI, depression, and MI + depression subgroups. *p<0.01, n=10 per subgroup. **B**) Quantification of 5-HT level in platelets. GFS pretreatment significantly increased 5-HT levels when compared with saline pretreatment for all subgroups: sham, MI, depression, and MI + depression. *p<0.01, n=10 per subgroup. **C**) Quantification of 5-HT level in the brain. With GFS pretreatment, 5-HT levels declined for all four animal subgroups. The declines for the MI-only (*p=0.025) and depression-only (*p=0.044) subgroups were significant, while the decreases did not achieve significance for the sham (*p*=0.060) and MI + depression subgroups (*p*=0.663).

## RESULTS

### Effect of GFS on 5-HT

At the end of the experiments, we measured 5-HT levels in the rat serum (Fig. 1A). When we compared 5-HT expression in the serum between saline- and GFS-pretreated animals for the four subgroups: GFS pretreatment increased 5-HT levels in the sham, but this did not reach a significant level (p=0.184). Instead, we report significant increases in 5-HT levels in the MI, depression, and MI + depression subgroups (all *p*<0.01 *vs.* respective saline pretreated counterparts). Figure 1B shows 5-HT expression in the platelet lysate between saline and GFS-treated animals for the four subgroups. GFS pretreatment significantly increased 5-HT levels for all subgroups when compared with saline pretreatment (all *p*<0.01). In the brain (Fig. 1C), GFS pretreatment caused 5-HT levels to decline in all four subgroups when compared with saline-pretreated animals. The declines for the MI (p=0.025) and depression subgroups (p=0.044) were significant, while the differences did not reach significance for the sham (p=0.060) and MI + depression subgroups (p=0.663).

**Figure 2.**
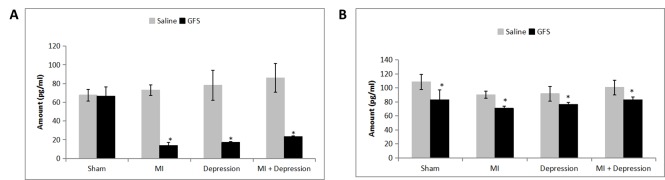
**5- HT_2A_R levels were measured in the platelets and brain tissues using an ELISA kit.** Data are presented as mean ± SD. **A**) Platelet 5-HT_2A_R quantification. There was no significant difference in 5-HT_2A_R levels between the saline- and GFS-pretreated groups in the sham subgroup (p=0.838). GFS pretreatment induced significant decreases in the MI, depression and MI + depression subgroups. *p<0.01 *vs.* saline, n=10 per subgroup. **B**) Brain 5-HT_2A_R quantification. All four subgroups demonstrated significant decreases in 5-HT_2A_R levels after GFS pretreatment. *p<0.01 *vs.* saline, n=10 per subgroup.

### Effect of GFS on 5-HT_2A_R

5-HT_2A_R was not measured in the serum because it is membrane-bound and not found in serum. There was no significant difference in platelet 5-HT_2A_R levels (Fig. 2A) between the saline and GFS pretreatment in the sham model (*p*=0.838). It was further indicated that GFS pretreatment decreased 5-HT_2A_R for the MI, depression, and MI + depression subgroups when compared with saline pretreatment subgroups (all *p*<0.01). In the brain (Fig. 2B), there was a significant decrease in 5-HT_2A_R levels in GFS-treated animals for all four subgroups (p<0.01) when compared with saline-pretreated animals.

## DISCUSSION

In the present study, we found that GFS have significant effects on expression of 5-HT and 5-HT_2A_R in MI, depression or both. GFS intervention reverses 5-HT declines in the serum of MI, depression and MI + depression rats. GFS pretreatment also elevates 5-HT protein levels in platelet from sham, MI, depression or MI + depression rats, reversing the decline seen in the saline-treated disease model. Further, GFS lowered 5-HT levels in the brains of MI-only and depression-only, but not MI + depression or sham rats. Surprisingly, 5-HT levels in the brain were decreased in GFS-pretreated MI-only and depression-only rats, which were not consistent with results obtained from the serum and platelet lysate. The implications of this finding however, are still unclear. Nonetheless, GFS did have a clear effect on 5-HT_2A_R levels in both the platelet and brain. To further evaluate the viability of platelets as a proxy for brain tissue in assessing 5-HT levels, the use of more sensitive techniques may be warranted.

It has been confirmed that 5-HT and its related pathway play important roles in the activation of platelets. 5-HT dysfunction is a key factor in the pathogenesis of depression [16, 34]. In the course of coronary heart disease, a variety of predisposing factors can lead to 5-HT secretion after platelet activation. 5-HT secretion mediated by 5-HT_2A_R induces platelet aggregation and coronary artery contraction. After 5-HT binds to 5-HT_2A_R on the platelet membrane, it is transported into the platelet through the serotonin transporter (SERT) and stored in dense granules. On the other hand, 5-HT levels decrease while platelet 5-HT_2A_R expressions increase during depression. Therefore, activation of the platelet 5-HT_2A_R could cause a more than usual increase in 5-HT signaling to result in increased platelet aggregation as well as alteration of platelet reactive activity. Such changes are similar to the platelet response in atherosclerotic disease. Patients with depression experience a decrease in 5-HT levels, an up-regulation in the 5-HT receptor, and functional decline of SERT resulting in reduced 5-HT re-uptake rate and inter-synaptic 5-HT concentration imbalance [35-37]. Our results suggest that GFS contributes to 5-HT stabilization by decreasing 5-HT_2A_R density.

In the clinical setting, serum levels of 5-HT are affected by many factors. This, along with other reasons makes the clinical feasibility of using cerebrospinal fluid to detect 5-HT relatively poor; brain biopsy is even more difficult in practice. However, the mechanisms for 5-HT uptake and release are similar for platelets and neurons in the CNS. Neurons and platelets also have significant structural and functional similarities thus, platelet 5-HT_2A_R has been used as a plausible proxy for CNS 5-HT_2A_R. Considering all these, we were led to choose platelet and brain tissue homogenates of 5-HT and 5-HT_2A_R as targets for our research. Our previous study found that rats with acute myocardial infarction, depression or a combination of the two showed significant elevation in platelet 5-HT_2A_R levels compared with the control group, with the combined myocardial infarction and depression group displaying the greatest increase [38]. This association of platelet 5-HT_2A_R with acute myocardial infarction showing symptoms of depression suggests a potential diagnostic tool for coronary heart disease and depression disorders, a comparatively sensitive and specific biomarker of the outer periphery [38]. Our results suggest, though, that the uptake and release of 5-HT between platelets and neurons is not equivalent.

Integrative Chinese and Western medical treatment has a therapeutic effect on coronary heart disease and depression. In Chinese medicine, ginseng has long been regarded as the king of herbs, nourishing vitality, improving stamina and generally contributing to well-being. Modern medical research is consistent with these traditional beliefs. Studies have shown that ginseng may improve cardiovascular blood flow, correct blood viscosity, enhance left ventricular function, and also significantly improve mental stress induced by myocardial ischemia [39]. Comorbidities of cardiovascular and psychological disorders are receiving increasing attention. Psycho-cardiology has become one of the hot spots of medical research. There is great promise that combination therapies may be proven to have certain advantages and efficacies. Our current study has shown the stabilizing effect of GFS on the serotonin system after depression, as well as myocardial infarction with depression, but not with acute myocardial infarction alone. One limitation of our study is that because we used total GFS, we were unable to identify the active ingredient with the largest effect as well as its related mechanism of action. For future studies, we plan on investigating ginsenoside monomers.

In conclusion, GFS has a clear role in modulating 5-HT expression and is effective in restoring 5-HT levels in MI and depression. Our findings warrant further in-depth study of the molecular mechanisms governing the body's response to GFS in various pathological states.
